# Identification of HMG-box family establishes the significance of SOX6 in the malignant progression of glioblastoma

**DOI:** 10.18632/aging.103127

**Published:** 2020-05-10

**Authors:** Lan Jiang, Hui Yang, Tianbing Chen, Xiaolong Zhu, Jingjing Ye, Kun Lv

**Affiliations:** 1 Central Laboratory, Yijishan Hospital of Wannan Medical College, Wuhu 241001, China; 2 Key Laboratory of Non-coding RNA Transformation Research of Anhui Higher Education Institution, Wannan Medical College, Wuhu 241001, China

**Keywords:** lncRNA, ceRNA, HMG-box, SOX6, glioblastoma

## Abstract

Glioblastoma multiforme (GBM) is the most malignant neuroepithelial primary brain tumor and its mean survival time is 15 months after diagnosis. This study undertook to investigate the genome-wide and transcriptome-wide analyses of human high mobility group box (HMG-box) TF (transcript factor) families / HOX, TOX, FOX, HMG and SOX gene families, and their relationships to GBM. According to the TCGA-GBM profile analysis, differentially expressed HOX, FOX, HMG and SOX gene families (62 DEmRNA) were found in this study. We also analyzed DEmRNA (HMG-box related genes) co-expressed eight DElncRNA in GBM, and constructed a ceRNA network analysis as well. We constructed 50 DElncRNA-DEmiRNA-DEmRNA (HMG-box related genes) pairs between GBM and normal tissues. Then, risk genes SOX6 and SOX21 expression were correlated with immune infiltration levels in GBM. SOX6 also had a strong association with MAPT, GSK3B, FYN and DPYSL4, suggesting that they might be functional members in GBM.

## INTRODUCTION

Glioblastoma multiforme (GBM) is the most malignant neuroepithelial primary brain tumor [1]. For GBM patients, its mean survival time is 15 months after diagnosis [2]. HMG-box (high mobility group box) domains are associated with the HMG-box proteins which influence DNA-dependent processes (transcription, replication, and DNA repair) and require changing the conformation of chromatin [3].

The HMG-box gene family is a family of TF-encoding genes which include a DNA-binding homeobox domain [4], such as HOX, FOX, SOX, HMG, and TOX genes. There were many studies on HMG-box genes in gliomas. HOX gene family was highly expressed in GBM cancer stem cells compared with parental lines, and HOX-PBX inhibition was a potential therapeutic target for GBM patients [5], and HOXD10 was targeted by hsa-miRNA-23a to inhibit glioma cell invasion [6]. Sex-determining region Y (SRY)-related high mobility group box of genes was abbreviated as SOX genes [7]. Using human glioma-initiating cell (GIC) lines (GIC1 and GIC2) created from anaplastic oligodendroglioma (AO) and GBM, both GIC1 and GIC2 expressed SOX2 and SOX3, and neither GIC line expressed SOX1 [8]. The gliogenesis of GBM was dependent on SoxD (SOX5, SOX6 and SOX7) and SoxE (SOX8, SOX9 and SOX10) [9]. SOX6 was specifically expressed by IgGs in GBM [10]. The moderate expression of SOX10 and SOX11 was linked to glioma, whereas the overexpression of them were associated with GBM [11]. SOX9 expression is connected to a poor prognosis of GBM patients and with resistance to temozolomide [12]. SOX2 / SOX21 axis could function as a tumor suppressor during glioma genesis [13]. However, SRY, SOX12, SOX15, SOX18, and SOX30 have not been reported to be associated with GBM. FOXM1 overexpression promoted clonogenic growth of GBM cells. FOXG1 and SOX2 via transcriptional control of core cell cycle and epigenetic regulators to fuel unconstrained self-renewal in GBM stem cells [14]. SOX9 and FOXG1 co-regulated a subset of EGFR [15]. Hsa-miR-338-5p also played a tumor suppressor role in glioma by binding FOXD1 [16].

Non-coding RNA plays an important role in post-transcriptional control of many animals [17, 18]. Numerous miRNAs could also bind and regulate SOX genes in GBM. SOX5 was over-expressed in GBM tissues, SNHG12-miR-195-SOX5 feedback loop could regulate the glioma cells’ malignant progression [19]. MiR-143, miR-253, miR-452 and miR-145 could down-regulate SOX2 in GBM, whereas miR-145 worked as a tumor-suppressive RNA by targeting SOX9 in human glioma cells [20]. SOX7 inhibited GBM tissue and was regulated by several miRNAs, such as miR-595 [21], miR-24 [22], miR-128 [23] and miR-616 [24].

However, transcriptomic- and genomic- wide systematic studies of HMG-box families in GBM is lacking. In order to better solve this problem, integrated analysis of HMG-box related gene family in GBM based on data gathered from GEO and TCGA database. We expected to find the DE-HMG-box and related non-coding RNA in GBM, and discovered the potential drug or disease target for GBM. Our findings provided new insights into the molecular role and phylogeny of the HMG-box families in GBM.

## RESULTS

### Transcriptomic identification of DEGs between GBM and normal tissues

**Figure 1:**
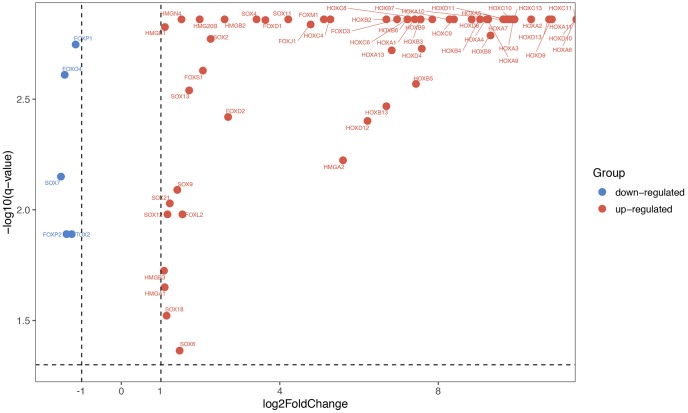
**Differentially expressed HMG-box genes were displayed via volcano chart.**

By obtaining data from TCGA database, we re-analyze the transcriptomic profiles of TCGA-GBM dataset, and 174 samples (169 GBM tissues and 5 normal tissues) were chosen to obtain DEmRNA (differentially expressed mRNA) and DElncRNA (differentially expressed lncRNA). GBM miRNAs expressed profiles were downloads from GEO database (GSE90603). There were 123 HMG-box genes that exist in TCGA-GBM. Through the analysis of the TCGA datasets, it was found that partial SOX, FOX, HOX, TOX and HMG gene families (a total of 62 genes) were significantly differentially expressed (|logFC| > 1 and q-value < 0.05) in our study, relative expression heatmap visualization was drawn in Supplementary Figure 1. Starting from the left, the first 5 datasets were normal tissues, and the remaining 169 were GBM tissues. Differentially expressed HMG-box genes were displayed via volcano plot (Figure 1). Only five HMG-box DEGs (FOXP1, FOXO4, SOX7, FOXP2 and TOX2) were downregulated between GBM and normal tissues, the others were up-regulated.

**Figure 2:**
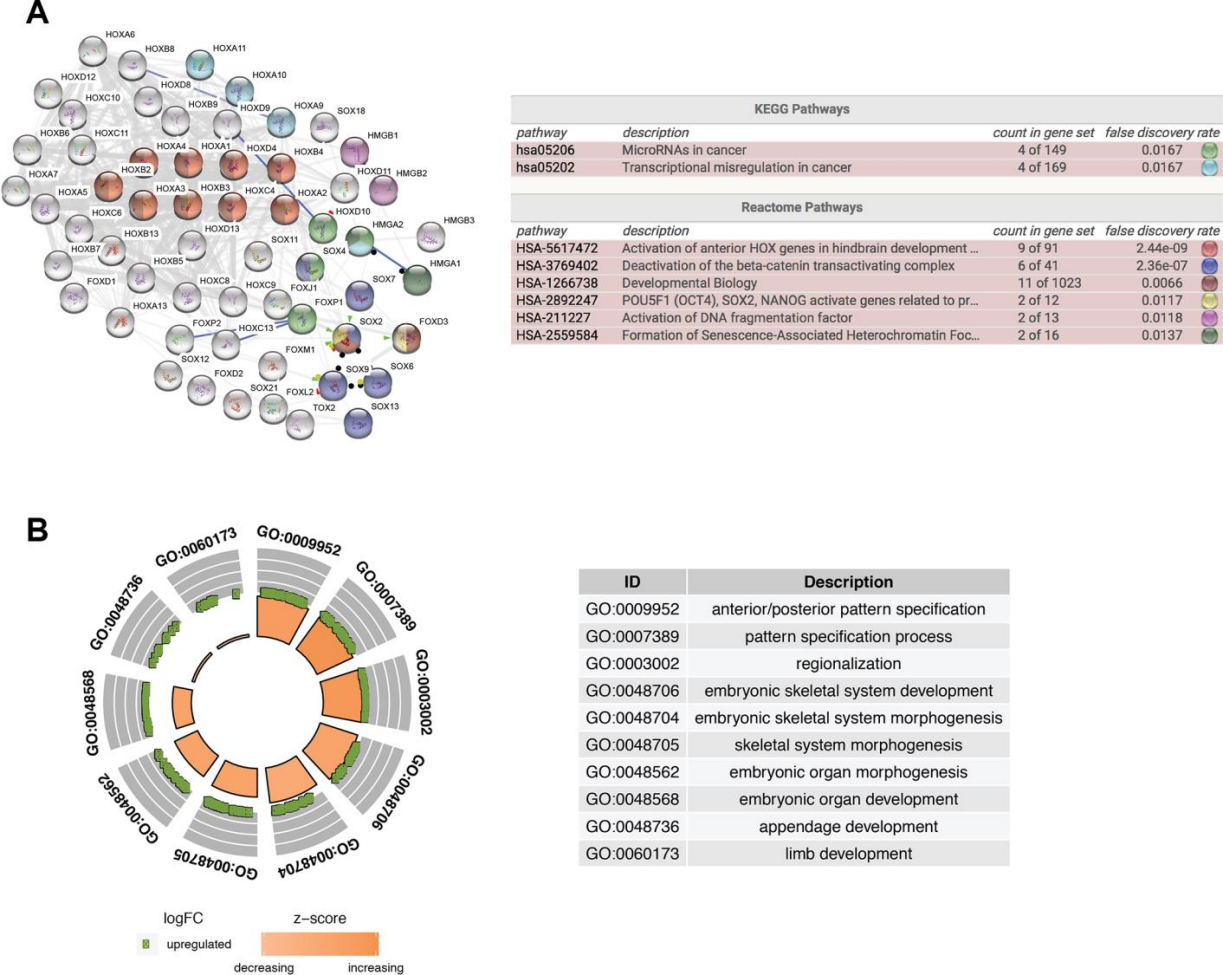
**Functional enrichment analysis of differentially expressed HMG-box related genes.** (**A**) Integrative analysis of PPI network and pathway enrichment analysis (KEGG and Reactome). (**B**) The top ten of GO enrichment analysis.

To further explore the function of isolated DE-HMG-box related genes, these 62 DEmRNAs were entered into clusterProfiler for GO enrichment. PPI network and Reactome and KEGG pathway enrichment analyses were built by STRING database and also presents the most significant enriched pathways of DE-HMG-box related genes in GBM (Figure 2A). Moreover, HMGA2, HOX9-11 were enriched in “Transcriptional misregulation in cancer” in the KEGG pathway. From Reactome pathway results, we found these genes enriched in six pathways, such as HOXA1-4, HOXB2-4, HOXC4, SOX2, and FOXD3 were enriched in “developmental biology”, HMGB1-2 were enriched in “Activation of DNA fragmentation factor” (Figure 2A). The top ten GO enrichment analysis results (q-value < 0.05) were shown in Figure 2B, the most significantly enriched in “GO:0009952: anterior/posterior pattern specification” (Figure 2B).

### Identification of HMG-box DEGs co-expressed lncRNAs

**Figure 3:**
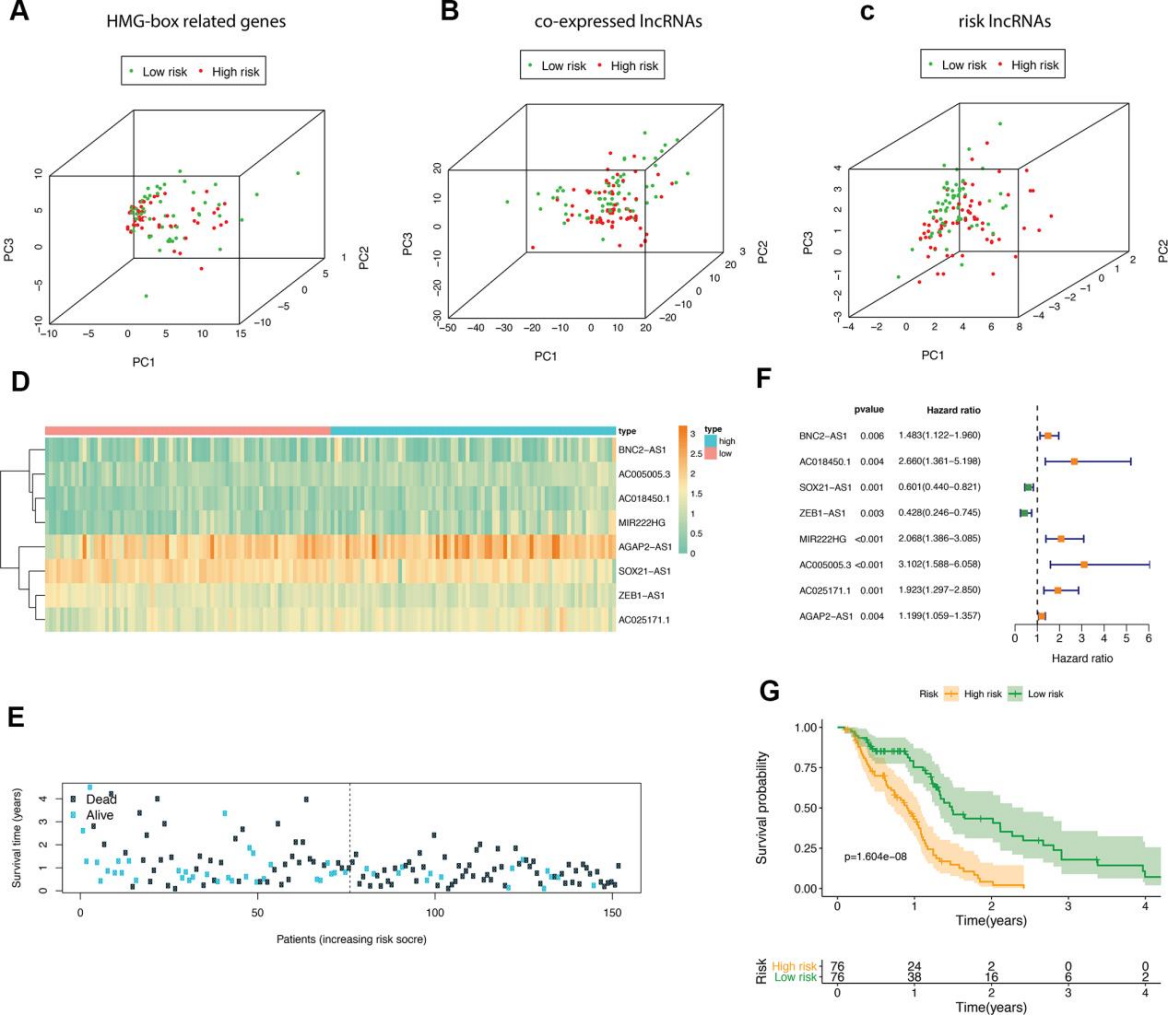
**The analyses of HMG-box related DEmRNAs co-expressed lncRNA.** Principle component analysis (PCA) of HMG-box related DEmRNAs was shown in (**A**), PCA analysis for co-expressed lncRNA in (**B**); PCA analysis for risk lncRNA was shown in (**C**). (**D**) Heatmap of risk lncRNA among high and low risk groups. (**E**) The distribution of co-expressed lncRNA survival status and survival time in model group. (**F**) Forest plot drawing for the independent prognostic value of risk lncRNAs extracted from univariate Cox regression analysis. (**G**) The survival curves of GBM patients in model group.

According to the median risk score, GBM patients in TCGA were divided into high- and low-risk groups. We performed principle component analysis (PCA) graphs on the HMG-box related DEmRNA, co-expressed DElncRNA and risk DElncRNA (Figure 3A–3C), green dots present low risk, and red dots present high risk in GBM patients. The eight HMG-box related lncRNAs heatmap employed in constructing the risk scoring model and survival information were shown in Figure 3D, 3E. The hazard ratio of eight risk lncRNAs is shown in the forest plot (Figure 3F). Of these eight lncRNAs, six were detected as high risk (BNC2-AS1, AC018450.1, MIR222HG, AC005005.3, AC025171.1, AGAP2-AS1, coefficient > 0), while two were supportive (SOX21-AS1, ZEB1-AS1, coefficient < 0). We also found that the overall survival time of patients in the high-risk group was lower than that in the low-risk group (p-value <1.604e-08, Figure 3G).

**Figure 4:**
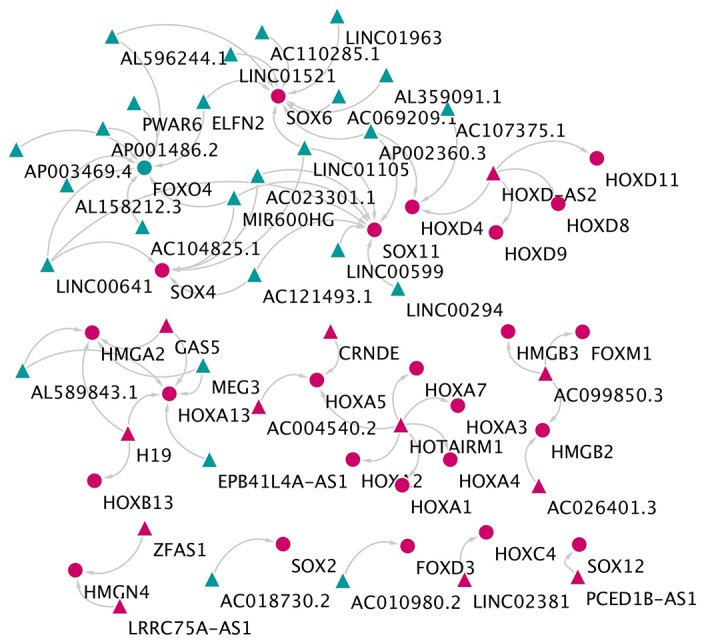
**The network of lncRNA and HMG-box related genes co-expression.** The triangles indicate lncRNAs, and circles mean mRNAs. The color green means down-regulated genes, and red means up-regulated genes.

A total of 147 DElncRNA (q-value < 0.05) were gained as well, of which 44 DElncRNA were up-regulated and 103 DElncRNA down-regulated. The association networks that included the DE-HMG-box gene families and their related co-expressed DElncRNA were constructed (Figure 4). The resulting lncRNA-mRNA association network had 68 interfaces between 38 lncRNAs and 27 mRNAs. The network showed that SOX6 was proposed to be the target of nine lncRNAs, FOXO4 was targeted by seven lncRNAs, and three mRNAs (HOXD4, SOX11, and SOX6) targeted AP002360.3.

### CeRNA network construction

We collected TCGA-GBM profiles (lncRNAs and mRNAs) and GEO data GSE90603 (miRNAs) in GBM through computational analysis to estimate potential relationships based on the ceRNA hypothesis to further understand their function. We found that 401 DEmiRNA (differentially expressed miRNA) is between seven normal tissues and sixteen GBM tissues (282 down-regulated and 119 up-regulated DEmiRNA). Using miRCode through miRNA response elements, eleven specific DEmiRNAs (three down-regulation and eight up-regulation) were detected to bind with sixteen DElncRNAs (fifteen down-regulation and one up-regulation).

**Figure 5:**
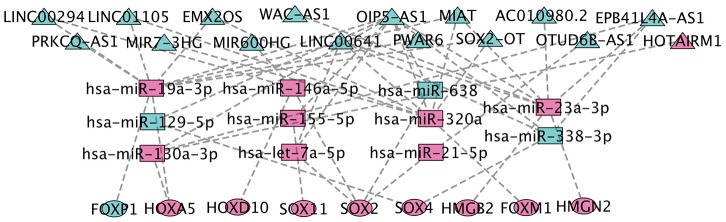
**CeRNA network construction.** The triangles represent lncRNAs, and circles mean mRNAs. The color green means down-regulated genes, and red means up-regulated genes.

In order to improve the prediction accuracy, TargetScan, SeedVicious and miRanda databases were combined to predict nine candidate DEmRNA targets for DEmiRNA. Cytoscape software was used to visualize a ceRNA network comprising sixteen lncRNAs, eleven miRNAs, and nine mRNAs based on the interactions between lncRNAs, miRNAs, and mRNAs (Figure 5).

### Risk score performance, comparison and combination of gene-signature

**Figure 6:**
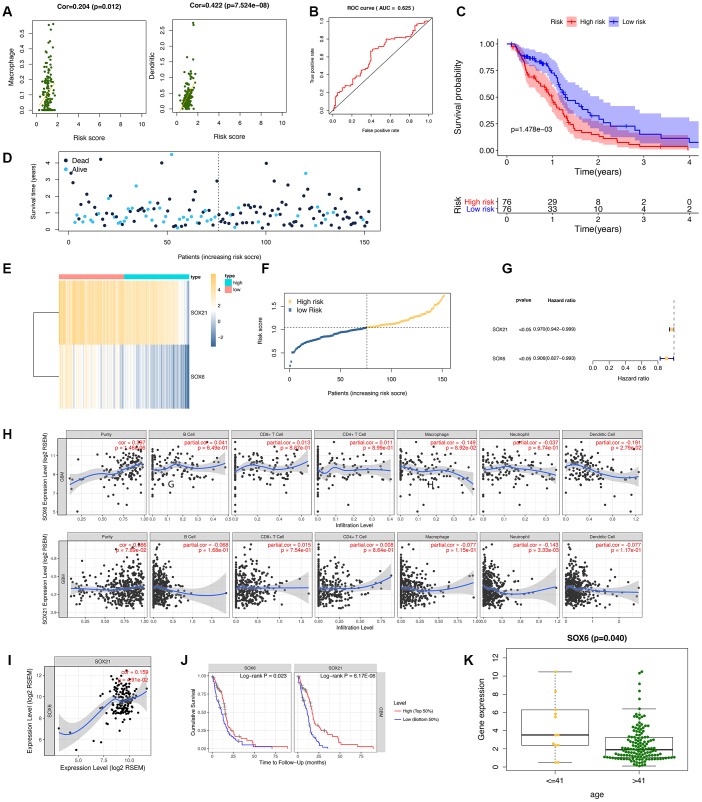
**Diagnostic studies and survival analysis in risk genes (SOX6 and SOX21).** (**A**) The correlation between the immune-infiltration abundance in risk genes (SOX6 and SOX21). (**B**) The receiver operating characteristic (ROC) curve for this model. (**C**) The survival curve is based on dividing the sample according to the median value of the risk value. (**D**–**F**) showed survival status of risk SOX6 and SOX21 among high and low risk groups. (**G**) Forest plot drawing for the independent prognostic value of risk HMG-box related obtained from univariate Cox regression analysis. (**H**) The correlation between the immune-infiltration abundance and the SOX6/SOX21 expression value. (**I**) The correlation of mRNA (SOX6, and SOX21) expression values in GBM by TIMER. (**J**) Survival curves of SOX6 and SOX21 in GBM. (**K**) The expression SOX6 between age of <=41 years and > 41 years.

To confirm the performance of the risk score in determining the survival rate of GBM patients, we used a model based on the prognostic dual genes (SOX6 and SOX21) signature to score the risk for each GBM patient. Risk genes (SOX6 and SOX21) expression levels were positively correlated with the infiltration levels of dendritic cells (p-value = 7.524E-08) and macrophages (p-value = 0.012) (Figure 6A). ROC curve analysis of five-year survival rate was used to evaluate the projection potential of two HMG-box-related genes. The area under the curve (AUC) of the prognostic model based on the properties of the two genes had a total survival time of 0.625 at 60 months (Figure 6B). Patients were categorized as high risk (n = 76) or low risk (n = 76), with the median risk being used as the cutoff value for survival analysis. Kaplan-Meier analysis showed that the overall survival curves of the two groups were significantly different (p-value = 1.478e-03, Figure 6C). Each patient's risk score (Figure 6D), survival status (Figure 6E), and spread of gene expression levels of both genes (Figure 6F) were also analyzed. In order to evaluate the performance of HMG-related genes as markers, we obtained two gene markers (SOX21, HR: 0.970 (95% CI: 0.942–0.999)); SOX6, HR: 0.906 (95%) to predict the prognosis of GBM patients through forest distribution maps. CI: 0.827-0.993)) (Figure 6G). Given the increasing association between immunological feature and prognosis in GBM cancer, we further explored the correlation between SOX6 and SOX21 in GBM. We explored whether SOX6 and SOX21 expression were correlated with immune infiltration levels in GBM. We measured the correlations of SOX6 and SOX21 expression with immune infiltration levels in GBM from TIMER. SOX6 expression level has significant positive correlations with infiltrating levels of purity, and significant negative correlation with dendritic cells in GBM, whereas the SOX21 expression level has significant negative correlation with neutrophil in GBM (Figure 6H). Subsequently, we further investigated the correlation between SOX6 and SOX21 gene expression in GBM patients, and the results showed that there was a significant positive correlation between SOX6 and SOX21 expression (Figure 6I). Regarding prognosis, Kaplan-Meier curves illustrated that GBM with SOX6-high had a worse prognosis than that with SOX6-low (p = 0.023), and with SOX21-high had a worse prognosis than that with SOX21-low (p = 6.17E-06) (Figure 6J). We also combined the clinical information to visualize the expression profiles of SOX6 and SOX21 and found that there is a significant difference in SOX6 only in terms of age composition (Figure 6K). These findings strongly implied that SOX6 might play a specific role in immune infiltration in different subtypes of GBM.

**Figure 7:**
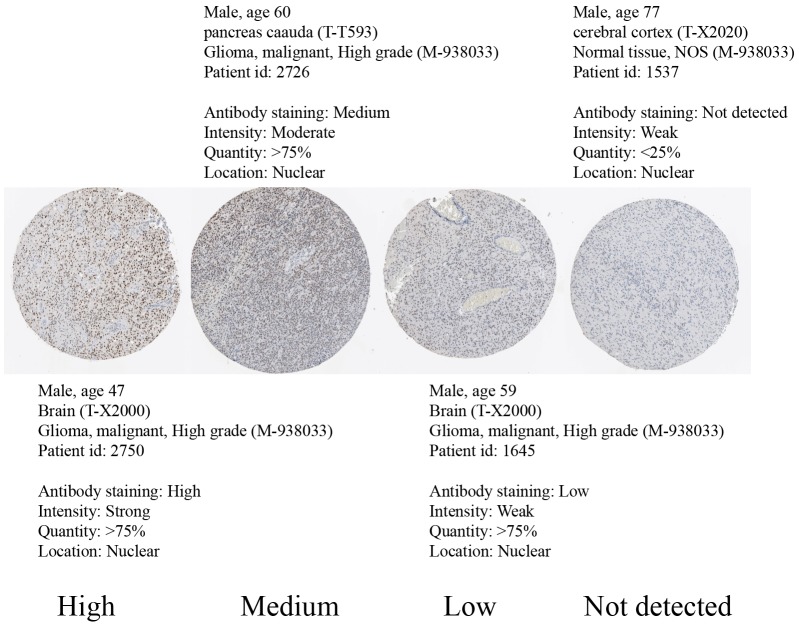
**Immunohistochemical staining of glioma tissue taken from the Human Protein Atlas showing SOX6-negative tissue (male, age 77) and high SOX6 (male, age 47), medium SOX6 (male, age 60), and low SOX6 (male, age 59) expressing glioma tissue.**

Data from the Human Protein Atlas database showed that immunohistochemistry staining of SOX6 protein was higher in GBM cancer tissue compared with normal tissue (Figure 7).

### Systematic analysis of SOX gene family and the importance of SOX6 in GBM

As a result, a total of 81 SOX members were identified in our study and divided into nine groups. Generally, the SoxA group contains SRY, SoxB1 has three members (SOX1, SOX2, SOX3), SoxB2 has two members (SOX14, SOX21), SoxC has three members (SOX4, SOX11, SOX12), SoxD contains SOX5, SOX6, SOX13, SoxE contains SOX8, SOX9, SOX10, SoxF contains SOX7, SOX17, SOX18, SoxH has SOX30, SoxG contains SOX15. As shown in Supplementary Figure 2A, the phylogenetic tree was constructed based on the SOX proteins using FastTree. We generated a graph to show the SOX protein structures by GSDS. According to the result of MEME suite, we found that there was a conserved and core motif (motif 1) in all SOX proteins, which is HMG-box domain and contains 79 amino acid residues (Supplementary Figure 2B and Supplementary Figure 2C). All motifs’ logos were shown in Supplementary Figure 2B and 2C. It’s noteworthy that motif 10, 2, 5 only appeared in SoxD group (SOX5, SOX6, and SOX13), they might be the domain to identify SoxD group. The SOX protein secondary structures showed in Supplementary Figure 2D and Supplementary Table 1. The secondary structures of SOX proteins were predicted by SOPM, PHD and PREDATOR methods on the NPS@, Network Protein Sequence Analysis website. For example, SOX6 was predicted to contain 38.12% α-helix, 4.45% β-sheet, and 54.89% random coil, respectively (Supplementary Table 1). By examining the properties of SOX genes for each of the four species (*Homo sapiens, Mus musculus, Coturnix japonica*, and *Gallus gallus*), the grand average of hydropathicity (GRAVY) value for those SOX genes mainly ranged from -1.080 - -0.206, which were higher than Mus musculus -1.984 - -0.207 (Supplementary Figure 2E and Supplementary Table 2). We found that the length of amino acids varied among species ranging from 204 - 817 nt. The distribution of molecular weight (Mol. Wt., kDa) for SOX genes ranged from 23.88 - 90.72. The isoelectric point (pI) of the SOX genes was from 4.91 - 9.96. According to the results of chromosome location, a total of 81 SOX gene members were mapped to the 14 chromosomes (Supplementary Figure 3).

**Figure 8:**
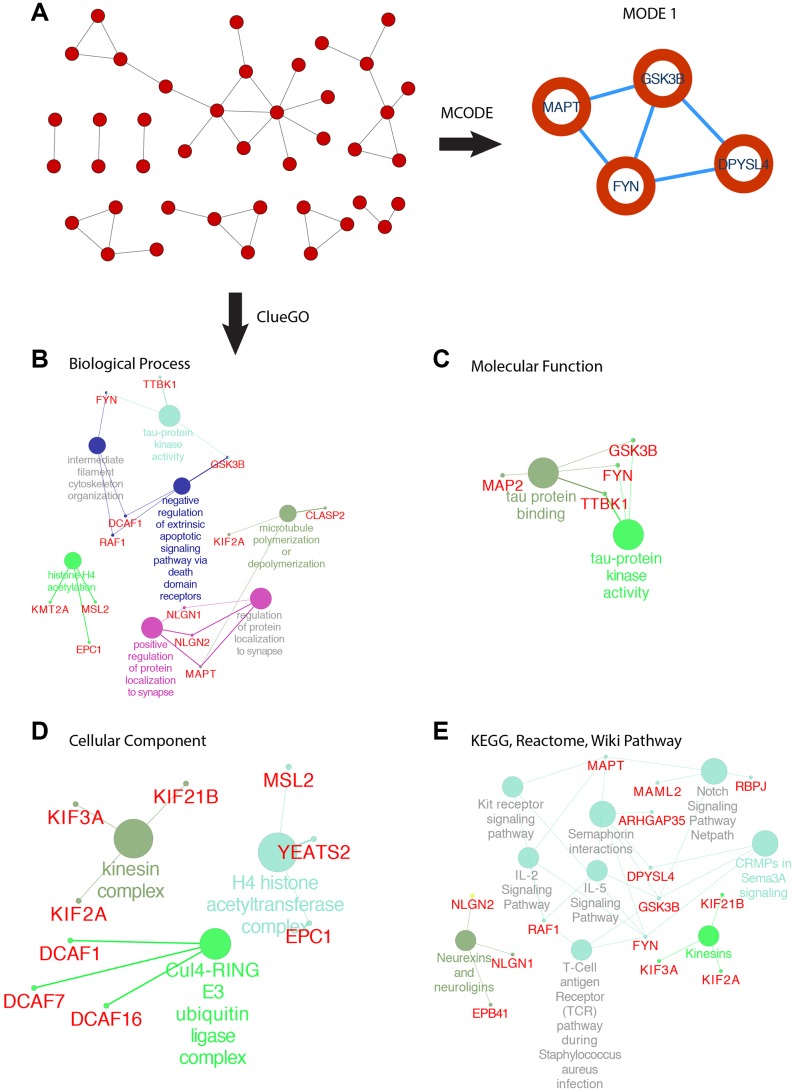
**PPI network of SOX6 positive correlation genes and functional analysis of hub genes.** (**A**) PPI network of SOX6 positive correlation genes and hub genes were found by MCODE in Cytoscape. (**B**) GO enrichment of co-expressed genes in biological process, (**C**) molecular function, (**D**) cellular component. (**E**) KEGG, Reactome, Wiki pathway enrichment analyses by ClueGO in Cytoscape.

SOX6 belongs to the SoxD group, based on the high expression in GBM, we used the GEPIA2 to obtain the top 200 co-expressed genes (Spearman’s correlation >= 0.68). Then, co-expressed genes network was constructed by STRING, and re-drawn by Cytoscape (Figure 8A). To analyze the biological classification and pathway of co-expressed genes, we used Cytoscape’s plugin ClueGO app for functional enrichment analyses (p-value <= 0.05). GO analysis indicated that the biological processes including tau-protein kinase activity (FYN, TTBK1, and GSK3B), intermediate filament cytoskeleton organization (FYN, DCAF1 and RAF1), negative regulation of extrinsic apoptotic signaling pathway via death domain receptors (DCAF1, RAF1 and GSK3B), histone H4 acetylation (KMT2A, MSL2 and EPC1), positive regulation of protein localization to synapse (NLGN1, NLGN2 and MAPT), microtubule polymerization or depolymerization (KIF2A and CLASP2) (Figure 8B). Consistent with enrichment of the respective cellular component and proposed molecular function (Figure 8C, 8D). Collectively, these data suggest an essential role of SOX6 in regulating cell survival and death mechanisms in GBM cancer.

**Figure 9:**
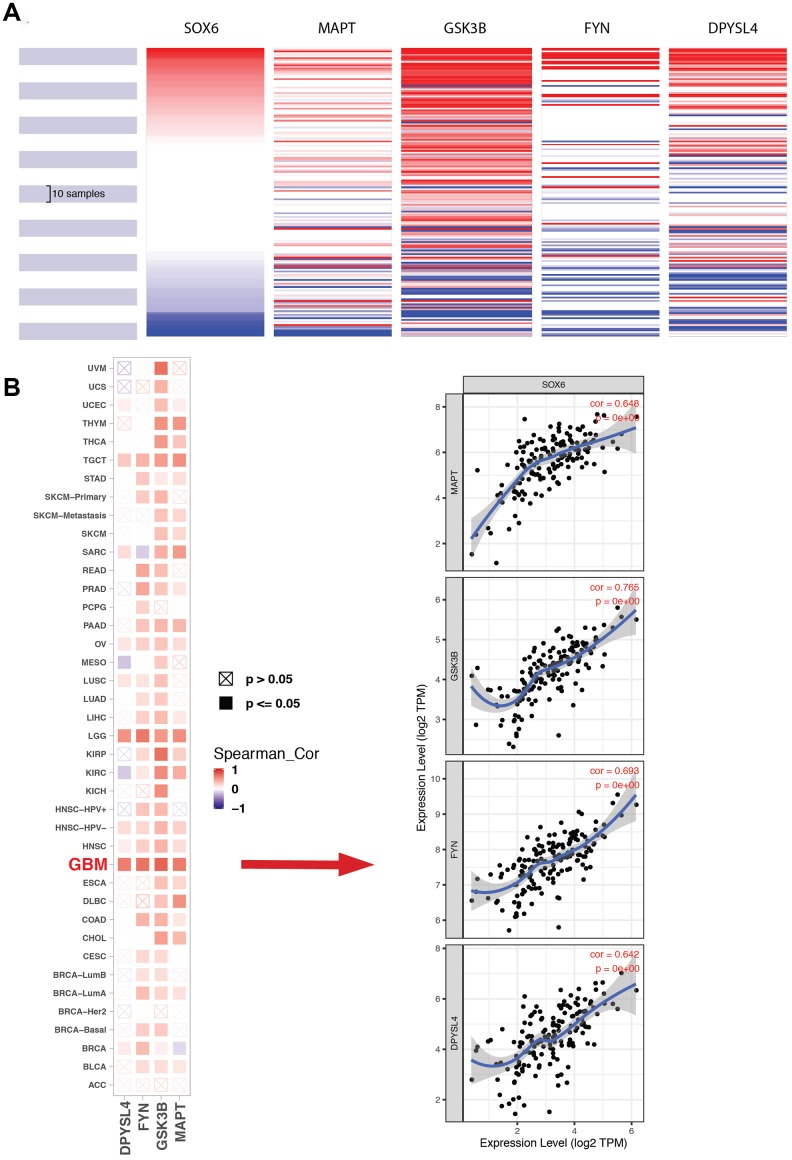
**Expression of SOX6 and hub genes.** (**A**) The hierarchical clustering of hub genes was generated by the UCSC online database. (**B**) The correlation between SOX6 and DPYSL4 (or FYN, or GSK3B, or MAPT) in different tumors, the correlation of co-expression in GBM was shown on the right by TIMER online browser.

The most important module was obtained using MCODE plugin (Figure 8A). We found MAPT, GSK3B, FYN and DPYSL4 as co-expressed hub genes. Hierarchical clustering of the hub genes was performed using the UCSC online tool (Figure 9A), indicating the concordant expression pattern across four genes. SOX6 compared with MAPT, GSK3B, FYN and DPYSL4 had the highest correlation coefficients (Spearman’s = 0.648, 0.765, 0.693 and 0.642) in GBM compared with other tumors (Figure 9B). This data demonstrated that SOX6 had a strong association with MAPT, GSK3B, FYN and DPYSL4, suggesting that they may be functional partners in GBM.

## DISCUSSION

GBM is an aggressive primary malignant brain tumor, and has one of the worst 5-year survival rates among all cancers after diagnosis [25]. It is the first time to report the role of HMG-box gene families and their related DElncRNA and DEmiRNA in GBM. Here, we presented a description of GBMs based on the integration of the genomic and transcriptomic profiles of the HMG-box gene families (such as SOX, HOX, FOX, HMG and TOX gene families).

Through significant differential expression analysis, we found that only 62 of the 123 HMG-box genes were significantly differentially expressed in GBM, and only five genes were down-regulated and 57 were up-regulated. From the expression distribution, this showed that most DE-HMG-box genes were specifically and highly expressed in GBM and had a very important role in enhancing cancer cells growth. From the PPI network and functional pathway results in this study, we found that partial HOX genes were correlated with transcriptional misregulation in cancer, activation of anterior HOX genes in hindbrain development during early embryogenesis, and developmental genetics. DE HMG-box families were closely linked to glioma-related tumors.

Mechanisms utilizing lncRNA have been shown to take part in various types of cancer. However, a comprehensive analysis of the differential expression profiles of DE HMG-box genes co-expressed lncRNA network in GBM has been lacking. Using multivariate Cox and risk score methods, we detected an eight-lncRNA signature which was able to classify GBM patients into the high-risk group and low-risk group with significantly different overall survival (p-value = 1.604e-08). Comparing our functional analyses with DE HMG-box genes, we discovered that eight-lncRNA might participate to GBM via development biology. Then, we further made a DE-lncRNA-DEmiRNA-DE-HMG-box network to expose the HMG-box related ceRNA mechanism in GBM. For example, in our ceRNA network, we found MIR200HG-hsa-miR-146a-5p-SOX2 / HOXD10 axis in GBM, down-regulated lncRNA MIR200HG could competitively bound miRNAs (up-regulated), thereby indirectly encouraging targeted SOX2 / HOXD10 upregulation. In HMDD (the Human microRNA Disease Database) v3.2 [26], we observed that the miRNAs are linked to glioblastoma or glioma, and found several connections in miRNA-mRNA binding. The upregulation of hsa-miR-146a and inactivation of NF-κB signaling induced the sensitization of human glioblastoma cells to TMZ-induced apoptosis by curcumin [27]. Hsa-miRNA-155 targeted FOXO3a could promote cell proliferation and invasion in glioma [28]. In this study, hsa-miRNA-155a-3p could combine OIP5-AS, and reduce the influence to SOX11, down-regulated MIAT, AC010980.2, and EPB41L4A-AS could target up-regulated hsa-miR-23a-3p, and formed a ceRNA network with up-regulated HMGB2 and HMGN2. As previously reported, Circ_PTN performed as sponge of miR-122, and activated SOX6 expression in glioma cells [29].

We systematically identified the HOX, FOX, SOX, TOX and HMG genes in humans, and also took SOX gene family as an example to comprehensive analysis of the SOX gene family from the phylogeny and protein structure. Although the absence of 3D structural data and the low accuracy of secondary structure prediction, we characterized secondary structures of SOX proteins to identify possible structural consequences of amino-acid substitutions, which indicated that these evolutionary changes have altered SOX protein function in some way.

In the current study, we initially screened out two DEmRNA (SOX6 and SOX21) of the SOX gene family that were found to be related to the clinical outcome of the GBM patients TCGA database. This study observed that up-regulated SOX6 could be targeted by eight down-regulated DElncRNAs in GBM tissues. SoxD group included SOX5, SOX6 and SOX13, we detected that SOX6 and SOX13 were over-expressed in GBM patients. SOX6 was expressed most frequently (6/7 or 86%) in GBM by RT-QPCR experiment [30], and expressed in the nuclei by immunohistochemical experiment [31]. GBM patients with low SOX6 expression present higher survival rates than those with high SOX6. FOXC1 could play the essential role in brain tumor biology and patients with GBM [32]. High expression of GATA2 connected with poor prognosis in GBM patients and promoted GBM progression by EGFR pathway [33]. SOX6 and SOX13 could co-interact with FOXC1 and GATA2, which might lead to aggressive the brain tumors. Hsa-miR-335-5p and hsa-let-7b-5p were significantly suppressed in GBM tissues [34], which targeted SOX13 in current study. We speculated that SOX6 might regulate GBM indirectly.

All the mechanism studies are aimed at finding drug targets, better prevention and treatment of diseases, there have been many studies such as a large number of alkylating anticancer agents and mutagens, and might be related to DNA replication [35–37]. As the HMG-box proteins influence DNA-dependent processes (transcription, replication, and DNA repair) [3], DE HMG-box genes and related DE-lncRNA / DE-miRNA in GBM, might serve as a potential drug target for DNA loss repair. Existing studies have shown that SOX5, SOX9 and SOX6 could be used as a drug target for INSULIN and DEXAMETHASONE, for the treatment of neurological diseases [38, 39], indicating that the combination of drugs and genes can reach the blood-brain barrier, so whether they could also be used as a drug target for solid tumor GBM, needs further research and mining by our research group.

## CONCLUSIONS

In summary, our study obtained the identification of HMG-box families and established a ceRNA network in GBM by TCGA and GEO dataset, presenting them as potential therapeutic targets for the treatment of GBM. Comparing our functional analyses with DE HMG-box genes, we identified that eight-lncRNA might contribute to GBM via development biology. SOX6 and SOX21 might represent a prognostic biomarker and potential therapeutic target to improve the diagnosis and treatment of GBM. SOX6 had a strong association with MAPT, GSK3B, FYN and DPYSL4 and might be functional partners in GBM. Our study provided useful information for further exploration of GBM. Moreover, more GEO datasets should be integrated to assess and reduce the bias during the analysis process, further experiments should validate in vitro and in vivo to make the role of these key genes and pathways clear in the development of GBM.

## MATERIALS AND METHODS

### Database selection and gene expression analysis of GBM

The gene expression datasets of GBM were downloaded from public database. (1) The Cancer Genome Atlas (TCGA) [40] (TCGA-GBM), which collected 169 GBM tissues and 5 normal tissues, were used to screen differentially expressed mRNAs (DEmRNAs) and differentially expressed lncRNAs (DElncRNAs) between GBM and normal tissues. (2) And the Gene Expression Omnibus (GEO) dataset GSE90603 (https://www.ncbi.nlm.nih.gov/geo/query/acc.cgi?acc=GSE90603), the platform for GSE90603 datasets was GPL21572, which contained 7 samples from non-tumor samples and 16 GBM tumor samples [41], were selected to analyze differentially expressed miRNAs (DEmiRNAs) between GBM and normal tissues.

Data analyses were performed by R packages, GEO dataset was downloaded by “GEOquery” [42] and TCGA-GBM data was gained from website, differentially expressed genes (DEG) were filtered out by “limma” [43], | logFC | > 1 and q-value < 0.05. “Ggplot2” [44] and “pheatmap” [45] were used to draw diagrams. Within the HMG-box DEGs, we performed the functional enrichment analysis of Gene Ontology (GO) function using “clusterProfiler” [46]. Survival analyses, the correlations with immune infiltration levels in GBM were performed by TIMER [47]. Immunohistochemical staining of glioma tissue extracted from the Human Protein Atlas [48].

### Analysis of DElncRNA-DEmRNA co-expression network

DElncRNA-DEmRNA (both genes with fold change > 2 and q-value < 0.05) co-expression network was built to determine the relationships in GBM (the absolute value of Pearson correlation coefficient > 0.5 and p-value < 0.001). The co-expression relationships were visually represented as the co-expression network using Cytoscape v3.7.2 [49].

### Single and multivariate factor cox analysis, ROC and survival curve plot

In order to predict the DEmRNA co-expressed lncRNA connected to survival, we performed the single factor Cox analysis by R package “survival” [50], risk model was calculated as previously reported [51]. According to the best risk model obtained by multivariate Cox analysis, the survival score was performed, and the average number of risk scores of each sample of TCGA-GBM data was also calculated. Above-average patients belong to the high-risk group, and below-average patients belong to the low-risk group. The Kaplan-Meier method was used to draw the survival curves of the two groups.

### CeRNA network construction

We then used DElncRNA, DEmiRNA and DEmRNA in this study to construct lncRNA-miRNA-mRNA associations, as previously reported [18, 52]. (1) DElncRNA-DEmiRNA interactions were described by miRcode 11 [53]. (2) Using 3’UTR regions for targeting, only DEmRNAs predicted by TargetScan [54], SeedVicious [55] and miRanda (score ≥150, MFE (minimum free energy) <−20 Kcal/mol) [56], were considered as target mRNAs, online software Venny v2.1.0 (https://bioinfogp.cnb.csic.es/tools/venny/) was used to scan the targeting DEmRNAs, and then performed the DEmiRNA-DEmRNA pairs. (3) According to the above DElncRNA-DEmiRNA and DEmiRNA-DEmRNA interactions, the visualization of the DElncRNA-DEmiRNA-DEmRNA network was built by using Cytoscape v3.7.2 [49].

### Example: Genome-wide retrieval and identification of SOX gene family

The genomic and protein data of human was downloaded from NCBI (ftp://ftp.ncbi.nlm.nih.gov/genomes/all/GCF/000/001/405/GCF_000001405.39_GRCh38.p13). Then, we downloaded the HMM file of HMG-box domain with InterPro ID (IPR009071) from Pfam v32.0 [57] and ran HMMER v3.2.1 [58] to obtain the SOX genes from the complete genome with e-value cutoff 1.8e-21 as previously reported [7].

SOX genes were mapped on chromosomes by Idiographica v2.4 [59]. The theoretical molecular weight (kDa), pI (isoelectric points), amino acids length and GRAVY (Grand Average of Hydropathy) values were evaluated using the ExPASy ProtParam platform (http://web.expasy.org/protparam/) [60], and then drawn violin plots by “easyGgplot2” [61] to illustrate the comparative relationship in human, mice, chicken and quail [62].

After finding and downloading SOX sequences, we used MAFFT v7.429 [48] to align the SOX genes, and constructed ML (maximizing the tree’s likelihood) tree by FastTree v2.1 [49]. Gene structures were drawn by GSDS v2.0 [50]. Motifs reported on SOX protein data via MEME v5.0.5 [51]. SOX secondary structure was built by Secondary structure by NPS@: Network Protein Sequence Analysis online service [52].

### Data mining for SOX6 and co-expressed hub genes

GEPIA2 (Gene Expression Profiling Interactive Analysis) [68] was used to analyze the gene expression correlation by TCGA-GBM data. The Spearman method was used to find the correlation coefficient. PPI network was constructed by STRING v11 [69]. Cytoscape’s plugin CluGO was used for functional enrichment analyses (GO, KEGG, Reactome, and Wiki pathway) [70]. Cytoscape’s plugin MCODE was applied for finding linked regions based on topology (MCODE score > 5, degree cutoff =2, node score cutoff = 0.2, max depth = 100, k-score =2). We plotted the heat map of SOX6 and co-expressed hub genes by University of California Santa Cruz (UCSC) browser [71], and the correlation of these genes was drawn by TIMER [47].

## Supplementary Material

Supplementary Figures

Supplementary Tables
