# Supplementary material for: Identification of HMG-box family establishes the significance of SOX6 in the malignant progression of glioblastoma
Source: Aging (Albany NY). 2020 May 10;12(9):8084–106. doi: 10.18632/aging.103127 (PMC7244032; doi:10.18632/aging.103127)
Supplement: Supplementary Figures [file aging-12-103127-s002..pdf]

SUPPLEMENTARY FIGURES

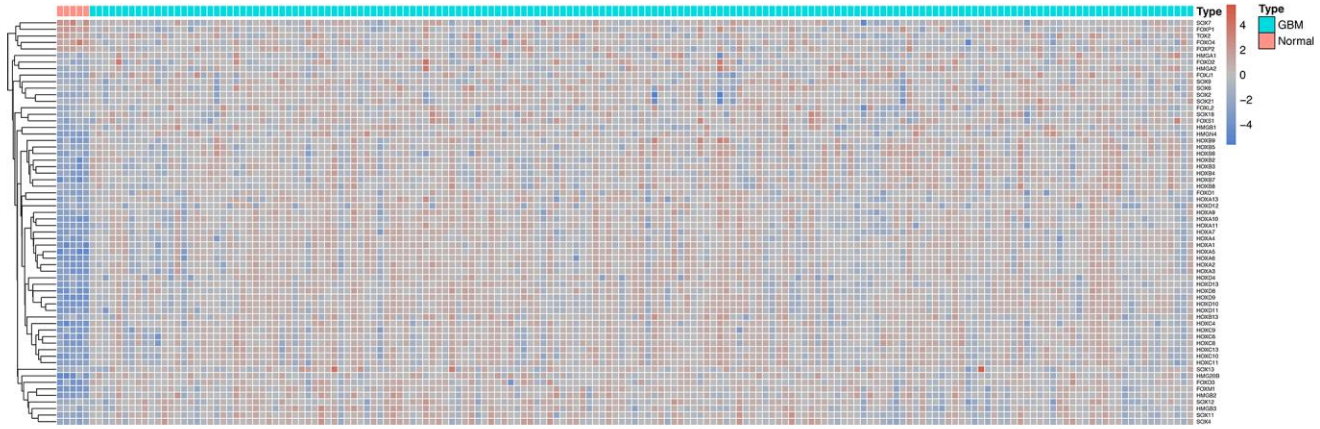

Supplementary Figure 1. Heatmap for HMG-box related genes between GBM and normal tissue in TCGA. Starting from the left, the first 5 datasets were normal tissues, and the remaining 169 were GBM tissues.

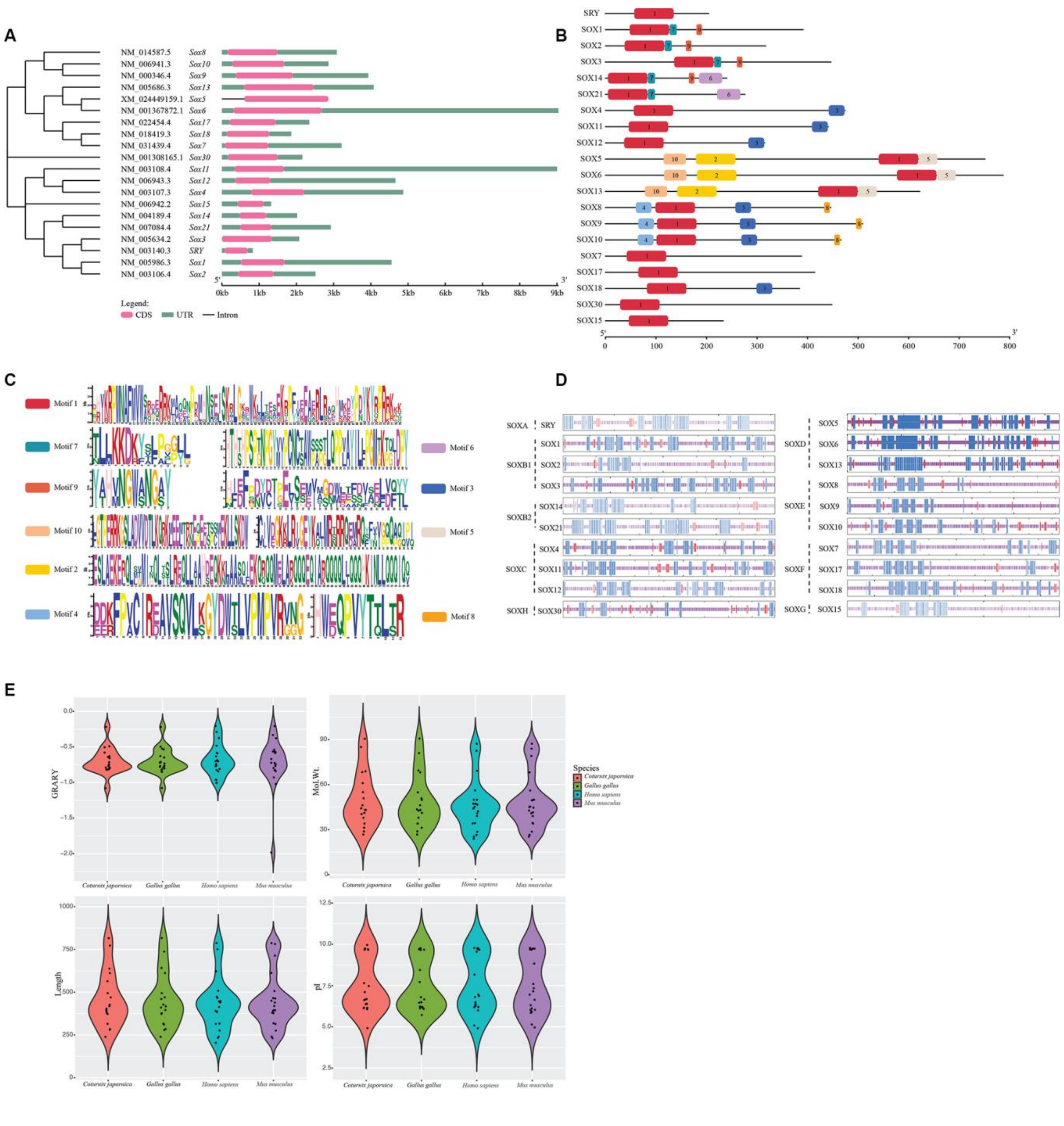

Supplementary Figure 2. Structures analyses of SOX gene family in humans. (**A**) Phylogenetic and protein structures analyses of SOX gene family in humans. (**B** and **C**) SOX motif prediction. (**D**) SOX protein secondary structures in humans. The α-helix, β-sheet and disordered loop regions are drawn in blue, red and purple, respectively. (**E**) Protein properties for SOX genes identified from *Homo sapiens, Mus musculus, Coturnix japonica*, and *Gallus gallus*.

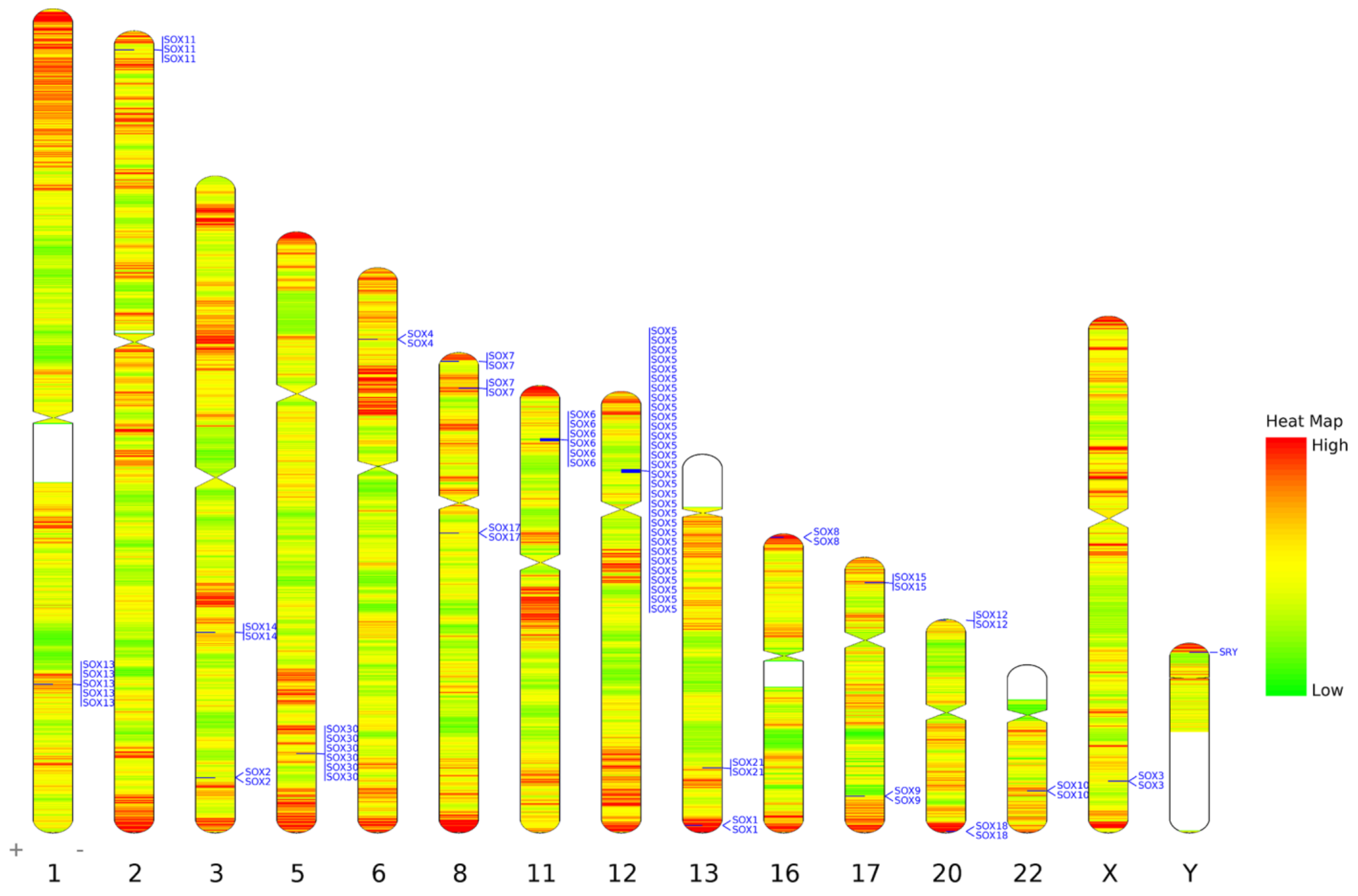

Supplementary Figure 3. Distribution of the SOX gene family on human chromosomes.
