# Supplementary material for: Identification of HMG-box family establishes the significance of SOX6 in the malignant progression of glioblastoma
Source: Aging (Albany NY). 2020 May 10;12(9):8084–106. doi: 10.18632/aging.103127 (PMC7244032; doi:10.18632/aging.103127)
Supplement: Supplementary Tables [file aging-12-103127-s001..pdf]

SUPPLEMENTARY TABLES

Supplementary Table 1. The composition of SOX protein secondary structures in humans.

Group	Type	Alpha helix (%)	Beta sheets (%)	Random coil (%)
SOXA	SRY	36.27	3.92	56.37
	SOX1	33.76	4.6	58.57
SOXB1	SOX2	15.14	3.15	79.18
	SOX3	31.39	3.14	62.33
SOXB2	SOX14	21.25	2.92	73.33
	SOX21	37.68	4.35	55.8
	SOX4	24.05	4.64	67.51
SOXC	SOX11	28.12	5.22	62.59
	SOX12	28.25	1.9	66.67
	SOX5	35.02	5.46	56.86
SOXD	SOX6	38.12	4.45	54.89
	SOX13	30.87	4.18	63.34
	SOX8	15.7	3.81	79.37
SOXE	SOX9	14.93	1.77	81.73
	SOX10	19.74	6.01	71.03
	SOX7	22.94	0.77	74.23
SOXF	SOX17	21.26	2.17	74.4
	SOX18	28.91	2.34	67.97
SOXH	SOX30	11.83	7.81	77.68
SOXG	SOX15	19.31	1.72	78.54

Supplementary Table 2. Predictions for protein properties for SOX from four species.

pI	GRAVY	Length	Mol.Wt.	Species
9.7	-0.375	391	39.02	*Homo_sapiens*
6.19	-0.825	466	49.91	*Homo_sapiens*
4.91	-0.702	441	46.68	*Homo_sapiens*
5.08	-0.96	315	34.12	*Homo_sapiens*
6.25	-0.766	622	69.23	*Homo_sapiens*
9.68	-0.585	240	26.49	*Homo_sapiens*
9.78	-0.843	233	25.25	*Homo_sapiens*
6	-0.633	414	44.12	*Homo_sapiens*
8.16	-0.589	384	40.89	*Homo_sapiens*
9.74	-0.742	317	34.31	*Homo_sapiens*
9.74	-0.206	276	28.58	*Homo_sapiens*
9.78	-0.29	446	45.21	*Homo_sapiens*
6.81	-0.701	448	49.88	*Homo_sapiens*
6.87	-0.483	474	47.26	*Homo_sapiens*
6.38	-0.745	751	82.58	*Homo_sapiens*
6.95	-0.809	787	87.24	*Homo_sapiens*
6.2	-0.687	388	42.20	*Homo_sapiens*
6.49	-0.77	446	47.31	*Homo_sapiens*
6.31	-1.007	509	56.14	*Homo_sapiens*
9.55	-0.968	204	23.88	*Homo_sapiens*
9.7	-0.379	391	39.05	*Mus_musculus*
9.74	-0.724	319	34.41	*Mus_musculus*
9.78	-0.332	450	45.44	*Mus_musculus*
7.15	-0.539	440	45.04	*Mus_musculus*
5.92	-0.767	714	79.13	*Mus_musculus*
6.95	-0.824	787	87.32	*Mus_musculus*
6.01	-0.671	380	41.49	*Mus_musculus*
6.64	-0.796	464	49.88	*Mus_musculus*
6.31	-1.021	507	56.08	*Mus_musculus*
6.12	-0.827	466	49.95	*Mus_musculus*
4.96	-0.727	395	42.63	*Mus_musculus*
5.14	-0.929	314	34.08	*Mus_musculus*
6.03	-0.745	613	68.17	*Mus_musculus*
9.68	-0.587	240	26.52	*Mus_musculus*
9.68	-0.842	231	25.31	*Mus_musculus*
5.85	-0.569	419	44.65	*Mus_musculus*
7.6	-0.565	377	40.90	*Mus_musculus*
9.74	-0.207	276	28.61	*Mus_musculus*
8.83	-0.575	782	83.94	*Mus_musculus*
7.34	-1.984	395	49.49	*Mus_musculus*
9.7	-0.495	373	37.93	*Gallus_gallus*
9.77	-0.78	312	34.10	*Gallus_gallus*
9.66	-0.712	316	34.01	*Gallus_gallus*
9.68	-0.63	240	26.67	*Gallus_gallus*
9.74	-0.22	280	28.80	*Gallus_gallus*
7.71	-0.65	428	43.11	*Gallus_gallus*

5.72	-0.818	396	43.50	*Gallus_gallus*
8.44	-0.72	285	31.26	*Gallus_gallus*
6.13	-0.733	737	80.99	*Gallus_gallus*
6.66	-0.825	817	90.72	*Gallus_gallus*
6.13	-0.801	612	68.18	*Gallus_gallus*
6.46	-0.788	470	50.83	*Gallus_gallus*
6.23	-1.078	494	54.85	*Gallus_gallus*
6.2	-0.851	461	49.86	*Gallus_gallus*
6.21	-0.524	410	42.60	*Gallus_gallus*
6.49	-0.766	418	46.23	*Gallus_gallus*
6.79	-0.769	377	41.22	*Gallus_gallus*
6.09	-0.536	642	69.47	*Gallus_gallus*
9.7	-0.505	373	37.92	*Coturnix_japornica*
9.96	-0.641	404	44.22	*Coturnix_japornica*
9.68	-0.715	316	34.03	*Coturnix_japornica*
9.68	-0.63	240	26.67	*Coturnix_japornica*
9.74	-0.218	280	28.77	*Coturnix_japornica*
7.1	-0.658	427	43.11	*Coturnix_japornica*
4.92	-0.77	396	43.18	*Coturnix_japornica*
7.65	-0.805	285	31.27	*Coturnix_japornica*
6.15	-0.746	773	85.08	*Coturnix_japornica*
6.66	-0.817	816	90.57	*Coturnix_japornica*
6.13	-0.81	612	68.32	*Coturnix_japornica*
6.37	-0.82	470	50.90	*Coturnix_japornica*
6.16	-1.08	495	55.04	*Coturnix_japornica*
7.47	-0.788	565	60.97	*Coturnix_japornica*
6.68	-0.58	387	40.39	*Coturnix_japornica*
6.4	-0.791	418	46.22	*Coturnix_japornica*
6.62	-0.759	377	41.16	*Coturnix_japornica*
6.07	-0.49	638	68.85	*Coturnix_japornica*
